# The association between patient’s compliance and age with the bonding failure of orthodontic brackets: a cross-sectional study

**DOI:** 10.1186/s40510-018-0209-1

**Published:** 2018-05-01

**Authors:** Isabela Vasconcelos Barbosa, Victor de Miranda Ladewig, Renata Rodrigues Almeida-Pedrin, Mauricio Almeida Cardoso, Joel Ferreira Santiago Junior, Ana Claudia de Castro Ferreira Conti

**Affiliations:** Rua Irma Arminda, 10-50, Bauru, Sao Paulo 17011-060 Brazil

**Keywords:** Motivation, Corrective orthodontics, Malocclusion

## Abstract

**Background:**

An efficient orthodontic treatment must aim the best occlusal result in the shortest possible time. One of the factors that can interfere in this goal is the bracket debonding during the treatment. This study aimed at assessing the different factors related to brackets failure, highlighting age and patient cooperation with treatment.

**Methods:**

The sample comprised 199 orthodontic patients of both genders (103 women and 96 men); divided into two groups—adolescents (12 to 18 years old, 118 patients) and adults (19 to 59 years old, 81 patients). A questionnaire was applied regarding the motivation of patients to seek treatment and whether they had received information on appliance care; patients also filled out their level of cooperation with treatment in a visual analog scale (VAS). Additionally, other variables were assessed, such as the teeth with bracket debonding, the presence of deep overbite, and the use of bite plate. The Mann-Whitney test was used, and a 5% significance level was applied for analyses.

**Results:**

It was observed that 20.1% of patients presented at least one tooth with bracket failure, and the lower arch was the most prevalent site (47.5%). Adolescents presented more debonding (25.4%) than adults (12.3%). Individuals with better VAS scores on cooperation sought treatment on their own (*p* = 0.042), were adults (*p* ≤ 0.001), and showed lower rate of failure of brackets (*p* ≤ 0.001). The factors related to malocclusion and treatment performed had no statistical significance.

**Conclusion:**

Greater cooperation was expected from adult individuals who sought treatment on their own and presented low rate of bracket failure.

## Background

All orthodontic advances of the last decade focus on optimizing the orthodontic treatment. The search for such efficiency, accounting for protocols for finishing treatment in less time, but also achieving the best possible outcomes have guided recent clinical researches. Besides the treatment duration, replacing brackets requires chair time and presents high cost, since it is not always possible to replace the same bracket [1–5]. This bracket failure may occur because of several factors, from occlusal trauma to inappropriate bonding techniques [1, 6–9].

Patient motivation for orthodontic treatment arises as an important factor, especially when considering time and even quality of the results. When the orthodontist cannot get the patients to adhere to treatment, they become less concerned and do not properly follow the instructions for appliances use and care, increasing the chances of appliance breakage thus compromising the treatment outcomes [1, 10–14]. Moreover, patients that are less committed to treatment and present higher bracket failure rate could have longer treatment duration, a situation that affects patient quality of life, considering financial and satisfaction aspects [2, 10, 12, 15, 16].

Nowadays, the search for shorter treatment duration is one of the main objectives of the health care system due to its implication on a better cost-benefit ratio [16]. In this context, the present research aims to identify the patient profile more susceptible to appliance breakage. In this way, the professional could establish more effective protocols to prevent this problem in those patients.

## Methods

This cross-sectional study was approved by the Research Ethics Committee of the (XXXX) University under protocol #1.090.493. The sample included 199 patients from 12 to 59 years old, 103 women and 96 men, during active phase of fixed orthodontic treatment with metallic brackets, from two post-graduation clinics and two private orthodontic clinics located in the same city, from August 2015 to July 2016. Those clinics were chosen because two faculty members worked in the same institution and they are the owner of the private clinics, so a standardized bonding technique was applied for all patients, which could minimize a potential source of bias. Patients from all ethnic groups under corrective orthodontic treatment in this age range who accepted to participate in the study were included. Patients presenting some cognition problem that could interfere with the adequate completion of the questionnaire were excluded. The main researcher (IBV) instructed and explained to the patients how to complete the questionnaire correctly and that the information would be confidential. The patients who agreed to participate in the research signed an informed consent form. The researcher followed patient care, verifying teeth and dental arches with failure of brackets; the presence and severity of initial overbite, which was classified when overbite value was higher than 3 mm; and the use of bite plate. These data were measured with the assessment of initial cast models of patients, performed with the help of a millimeter ruler.

The wide range of patient’s age aimed at evaluating the influence of age on bracket failure. Based on that, the patients were divided regarding age, and two groups were considered: adolescents (12 to 18 years old) and adults (19 to 59 years old).

A questionnaire composed by five questions was applied by the same researcher (IBV) without any interference for each patient as follows:

1. How old are you:
2. Mark your gender: a) Male b)Female
3. Did you seek orthodontic treatment: a) By your own initiative; b) Referred by another dentist; c) Because of a friend or relative
4. 4-When you started treatment did you receive any information about your diet in order to prevent brackets failure? a) yes; b) no; c) I don’t know
5. Do you consider yourself a collaborating patient following the instructions to avoid brackets failure? a) yes; b) no; c) I don’t know

Another parameter assessed was the phase of orthodontic treatment (in months) of the patient at the moment of assessment relative to the initial date of the corrective treatment. The patients were also instructed to report their level of cooperation with treatment, which was assessed by means of the visual analog scale (VAS). This scale was defined as a 100-mm line, where zero at the leftmost end indicated the less cooperation level, and the opposite end, on the right, indicated the best possible cooperation. The patients were instructed to mark a vertical stripe between the left and right ends of the line to indicate their scores of cooperation.

The overall bracket failure rate of the whole sample was assessed. These data regarding bracket failure rate was associated with patients’ age, gender, reported level of cooperation, and the patient motivation to seek treatment. Besides that the bracket failure were also analyzed according to the dental arch more affected and the presence or absence of deep overbite and the use of bite plate.

### Statistical analysis

Data obtained were organized in an Excel table (Microsoft Office Excel, Redmond, WA, USA) and were subjected to the SigmaPlot software (SigmaPlot, San Jose, CA, USA) version 12.0. The Shapiro-Wilk test was applied to verify wheter data presented normal distribution. As they did not, the Mann-Whitney test was used. For all statistical analyses, a 5% significance level was adopted.

## Results

Power analysis showed that a sample size of at least 199 patients would give an 100% (α = 1.0) probability of detecting a real difference between groups: mean 11.65 or median 16.4 (based on the scores from VAS regarding treatment cooperation and bracket failure) at a statistically significant level of 5%.

From 199 patients, 40 (20%) presented failure of brackets (Table 1). The lower arch was more affected than the upper arch (9.5 and 7.5%, respectively). Additionally, 3% of individuals assessed presented failure of brackets in both arches (Table 1).

**Table 1:** Descriptive statistics of bracket debonding incidence

Bracket debonding	*N*	%
Upper	15	7.5
Lower	19	9.5
Both	6	3.0
No debond	159	79.9
Total	199	100.0

An analysis on treatment sites in educational or private institutions found no significant difference in the effect of bracket breakage by patients, *p* = 0.499. Similarly, the presence or absence of deep overbite (*p* = 0.922) and the use of bite plate (*p* = 0.908) were not statistically significant (Table 2).

**Table 2:** Descriptive statistics of bracket debonding incidence by treatment place, overbite, and use of bite plane

	Variables					
	Treatment place^1^		Overbite^2^		Bite plane^3^	
	Private office	Orthodontic school	> 3 mm	< 3 mm	Yes	No
*N*	14	26	13	27	5	35
%	35.0	65.0	42.5	57.5	12.5	87.5

^1^*p* = 0.499

^2^*p* = 0.922

^3^*p* = 0.908

There was also no significant difference in the failure of brackets when considering patient gender, *p* = 0.097 (Table 3).

**Table 3:** Comparison between patients’ gender and bracket debonding

Gender		*N*	%	*p* value
Female		16	40,0	
Male		24	60,0	
Total		40	100.0	*p* = 0.097

On the other hand, an analysis on whether age (adolescent or adult) could influence bracket breakage showed that adolescents presented more breakage than adults, and the comparison was statistically significant, *p* = 0.02 (Table 4).

**Table 4:** Comparison between patient age, bracket debonding, and self-report of treatment cooperation

Age	*N*	%	VAS	*p* value
Teenagers (12 to 18 years)	30	75.0	64,5	
Adult (19 to 59 years)	10	25.0	82,2	
Total	40	100.0	100.0	*p* = 0.02*

*Significant association in 5%

**Fig. 1:**
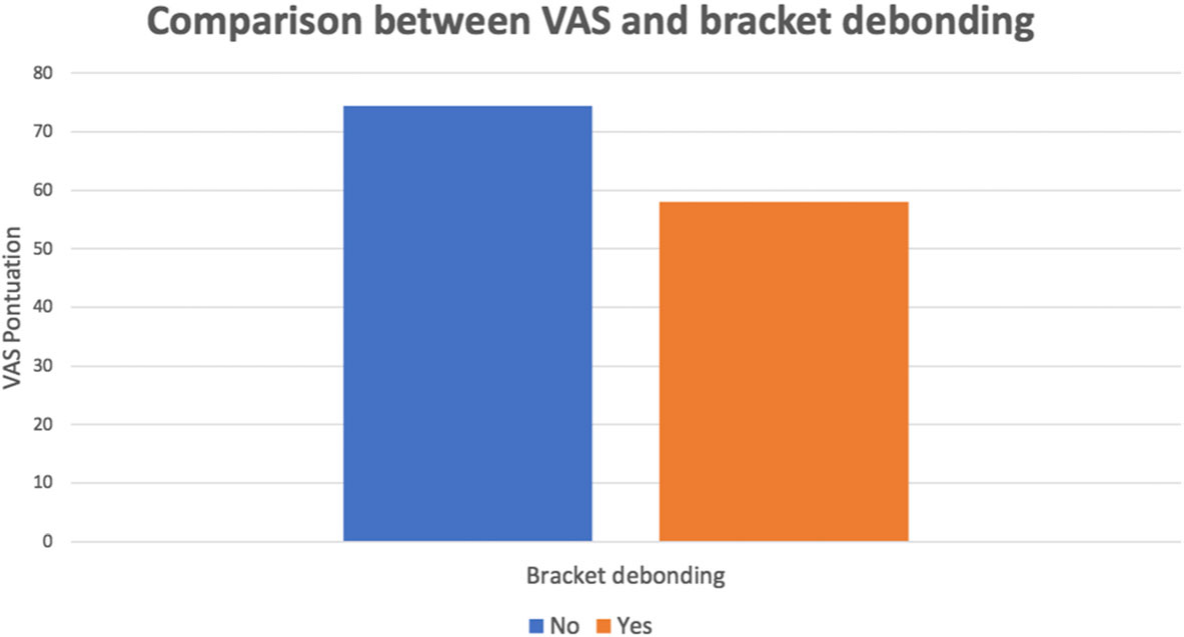
Comparison between patient self-report of treatment cooperation (VAS) and bracket debonding (*p* ≤ 0.001)

Regarding cooperation, which was investigated as a self-report of patients on the VAS score, it was found that the group without bracket failure presented the highest score (median 74.4) when compared to the group with breakage (median 58.00), *p* ≤ 0.001 (Fig. 1).

**Fig. 2:**
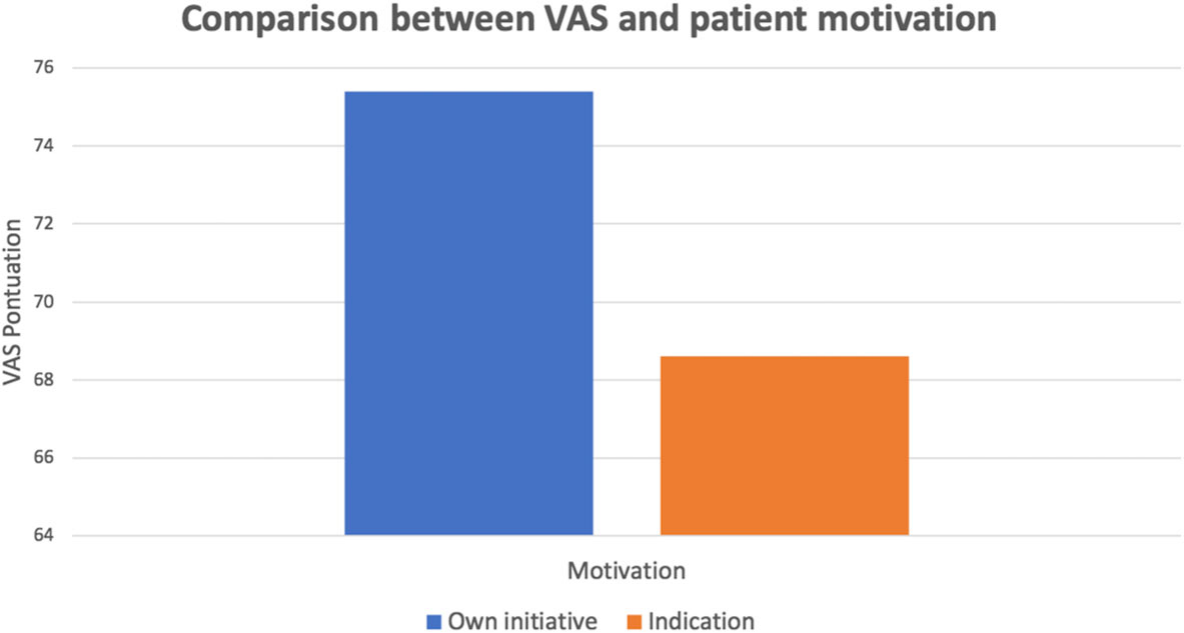
Comparison between patient self-report of treatment cooperation (VAS) and patient’s motivation for seeking treatment (*p* = 0.042)

An analysis of patient motivation (own initiative or referred by others) found no significant difference regarding bracket breakage, *p* = 0.596 (Fig. 2). However, a significant difference was identified for the VAS (*p* = 0.042), indicating that the group that sought treatment on their own presented higher scores (median: 75.4) than the group referred to orthodontic treatment (median 68.6).

Still regarding the VAS, it was observed that adult patients presented higher scores (median: 82.2) than adolescents (median 64.5), with *p* ≤ 0.001, showing that adults report themselves as more cooperative with treatment than younger individuals.

## Discussion

Patient motivation represents an important factor for orthodontic treatment success, and it is directly associated with patient cooperation regarding care and hygiene instructions provided by the orthodontist. A high level of motivation may decrease brackets failure, a common problem observed in the orthodontic practice that may causes great treatment delays [2, 10, 13, 15–17]. Robb et al. (1998) [15] reported that treatment duration changes up to 46% are related to bracket failure. Literature showed that an increase of 0.3 to 0.6 times in treatment duration could be attributed to each bracket failure [12, 18].

Understanding the type of patients who mostly present bracket breakage and the most prevalent arches and tooth could help the orthodontist to prevent this situation. Therefore, this research included an extensive sample of 199 individuals, which allowed observing the profile of patients who presented more failures during orthodontic treatment, as well as the most affected teeth.

It was found that 20% of patients from the sample presented some bracket breakage at the moment of assessment (Table 1). The literature shows a great variation in the prevalence of failure—from 3.5 to 23%—due to the different variables that may be considered [1, 16–18].

When assessing distribution per arch (Table 1), we found a higher bracket failure rate in the lower arch (9.5%), and this result agrees with previous studies [1, 5, 9, 19, 20]. The challenge in maintaining the lower arch dry during bracket bonding, the higher initial crowding, and the occlusal interference may be the causes of greater failure [1, 17, 20].

Overall, considering the areas and teeth more susceptible to breakage and failure, the professional may perform carefully the bonding procedure in these areas, preventing contamination with saliva and placing the bracket without occlusion interference.

In order to verify the level of patient cooperation regarding the instructions received, the VAS was used. A simple method that is easily understood and applicable by evaluators [21–24]. It was observed that the group of patients that presented bracket breakage (Fig. 1) showed a lower VAS score (median 58.0) than the group without breakage (median 74.4). These data are in accordance with the literature, which states that the number of brackets lost during treatment is inversely related to patient cooperation [10, 12].

Motivation becomes an important factor for great treatment outcomes. This characteristic may be observed even before therapy, when the individual decides to initiate treatment. This motivation may be described as either external, resulting from the pressure from friends or family members, or internal, resulting from a personal desire. This definition is important considering that it is suggested that patients internally motivated are more cooperative [14, 25].

In this research, when assessing patient motivation, we found that the group that sought treatment on their own presented higher VAS scores than the group that was referred by others (Fig. 2). This result could justify the fact that adult patients present lower breakage rate, because normally they seek treatment on their own, while adolescents do it by the indication of parents or other professionals [25].

In addition, some authors have observed a reduction in bracket failure and improvement in treatment efficiency when interventions with the purpose to improve patient compliance during orthodontic treatment have been implemented [26].

Considering the age group, it was observed that adult patients presented higher level of cooperation (median 82.2) than adolescents (median 64.5). A study performed in 2009 reported a correlation between patient motivation and level of cooperation during orthodontic treatment, and the most motivated patients were the ones that better followed the instructions provided by the orthodontist [13]. Accordingly, it was reported that 52% of adolescent patients do not follow the treatment instructions provided by the professional [11].

The aforementioned results could justify the fact that adult patients presented a lower breakage rate (12.3%) than adolescents (25.4%) (Table 4). These values agree with several studies that observed that the incidence of failure of brackets seems to decrease with age and that the level of cooperation of adults is higher, counterbalancing even the greatest mechanical difficulties in their tooth movements [1, 13, 16, 17, 25, 27, 28].

A limitation of our study comprises the evaluation in one time point, although the distribution of patients at various stages of orthodontic treatment could provide an overview of device breakages. In a future study, a long period of evaluation should be performed in order to confirm these findings.

Based on the information above, identifying patients who need to be more motivated along the treatment is a task professionals should not neglect. Spending some time encouraging and motivating patients to increase their level of cooperation with treatment is as important as a good treatment plan and execution.

## Conclusions

Adult patients, individuals who sought treatment on their own and those that considered themselves more cooperative presented lower rate of brackets failure.

Thus, means of motivating patients, especially adolescents, should be implemented to increase their cooperation and optimize the orthodontic treatment.

## Abbreviation

VAS: Visual analog scale
